# Dietary Inflammatory Index, Sleep Duration, and Sleep Quality: A Systematic Review

**DOI:** 10.3390/nu16060890

**Published:** 2024-03-19

**Authors:** Christle Coxon, Jun Nishihira, Piril Hepsomali

**Affiliations:** 1 School of Psychology, University of Roehampton, London SW155 4JD, UK; christle.coxon@roehampton.ac.uk; 2 Department of Medical Management and Informatics, Hokkaido Information University, Ebetsu 069-8585, Japan; nishihira@do-johodai.ac.jp; 3 School of Psychology and Clinical Language Sciences, University of Reading, Reading RG6 6ET, UK

**Keywords:** sleep, dietary inflammatory index, nutrition, systematic review, inflammation

## Abstract

The inflammatory potential of the diet, as measured by the Dietary Inflammatory Index (DII^®^), has been repeatedly shown to be associated with various inflammatory markers and mental and physical health outcomes. Of specific importance, several cross-sectional studies revealed mixed results regarding the correlations between the DII and sleep outcomes. Hence, in the current paper, a systematic review that examines the associations between the DII, sleep duration, and sleep quality was performed. The PubMed database was systematically searched for studies published up to November 2023 following PRISMA guidelines. Only cross-sectional studies that assessed the DII, sleep duration, and sleep quality across healthy and unhealthy cohorts were included. Eleven and seven studies were included in the systematic review for sleep quality and duration, respectively. The results of the present systematic review show that pro-inflammatory diets may be associated with poor sleep outcomes (duration and quality); however, as the current literature is inconsistent and limited, further cross-sectional studies in larger cohorts are necessary to (i) explore this relationship to address this heterogeneity and (ii) explore populations that are more sensitive to diet-induced inflammation.

## 1. Introduction

Poor diet, a leading risk factor for noncommunicable diseases, is linked to poor sleep outcomes [1]. For instance, previous studies have shown the benefits of fats; group B vitamins, magnesium, and l-tryptophan; foods containing tryptophan, melatonin, and phytonutrients(e.g., cherries, kiwifruit, milk) [2,3,4]; and dietary patterns such as the Mediterranean-style diet [5] and/or a high-quality diet [6] on sleep duration and sleep quality.

The aforementioned nutrients, food groups, and dietary patterns are also known to be associated with reduced systemic chronic inflammation (SCI) biomarkers, including platelet and leukocyte counts, neutrophil-to-lymphocyte ratios (NLRs), and C-reactive protein (CRP) levels [7,8,9,10]. For instance, large-cohort studies in healthy adults from Italy, the UK, and the US, assessing diet using food frequency questionnaires, found that adherence to a Mediterranean dietary pattern or higher healthy dietary scores was associated with lower markers of systematic chronic inflammation [7,8,9]. Additionally, a meta-analysis of five cross-sectional studies conducted in older adults (≥64 years)) revealed significant inverse associations between adherence to a Mediterranean dietary pattern and circulating CRP [10]. Moreover, evidence from meta-analyses of randomized controlled trials across various age groups have further supported the cross-sectional evidence above by showing reductions in CRP, interleukin-6 (IL-6), and interleukin-1 β (IL-1β) [11,12].

Additionally, an increasing body of research has highlighted connections between sleep patterns and SCI. For instance, previous studies have noted associations between (i) higher leukocyte counts and shorter sleep duration (<8 h/night) [13], (ii) higher levels of CRP and IL-6 and shorter sleep duration (<5 h/night) [14], and (iii) higher CRP levels, platelet counts, and NLR, and poorer sleep quality [8,15,16] was observed. Similarly, the inflammatory potential of the diet, namely the Dietary Inflammatory Index (DII^®^: a literature-derived estimation that is associated with circulating inflammatory biomarkers) [17] has also been shown to be related to sleep outcomes, such that more pro-inflammatory diets were correlated with shorter and longer sleep durations and lower sleep quality [18,19,20,21,22,23,24]. However, other studies observed null findings [25,26,27,28,29,30].

As evidenced above, the findings remain highly inconclusive, and to the best of our knowledge, this area of research has not been systematically reviewed. Despite the heterogeneity of the studies included in this review, our objective was to carry out a systematic review and assess the strength of the scientific evidence supporting the associations between sleep outcomes (namely sleep duration and sleep quality) and the DII.

## 2. Methods

### 2.1. Study Selection

The inclusion criteria were (1) the DII, a literature-based dietary score that quantifies the inflammatory potential of diet [17], being measured by 24-hour dietary recall, dietary survey data, or a food frequency questionnaire; (2) sleep duration or sleep quality, assessed by both subjective and objective measures; (3) cross-sectional studies; and (4) healthy or unhealthy (sleep and/or metabolic disorders) participants of any age or gender.

The exclusion criteria included (1) randomized controlled and quasi-experimental trials, case reports, letters to editors, conference papers, theses, personal opinions or commentaries, and (2) animal, in vitro, and ex vivo studies.

### 2.2. Search Strategy and Data Sources

An electronic literature search was carried out on PubMed and Scopus in order to identify appropriate studies. The literature search was conducted until the beginning of November 2023. The search strings used in the search included (DII OR “dietary inflammatory index” OR “inflammatory diet” OR “anti-inflammatory diet”) AND (sleep*). Manuscripts were selected according to the Preferred Reporting Items for Systematic Reviews and Meta-Analyses (PRISMA) diagram [31,32]. Papers were selected independently by one reviewer (PH) based on the inclusion and exclusion criteria specified above. Information regarding (1) Publication details (authors, year, journal), (2) Participant characteristics (number of participants recruited, number of participants included in the study, gender, age range, and health status), (3) Study design, (4) Measures (DII, sleep duration, and sleep quality), and (5) Notes (factors that might affect results and/or data quality) were extracted from all publications. 

## 3. Results

### 3.1. Study Characteristics

**Figure 1:**
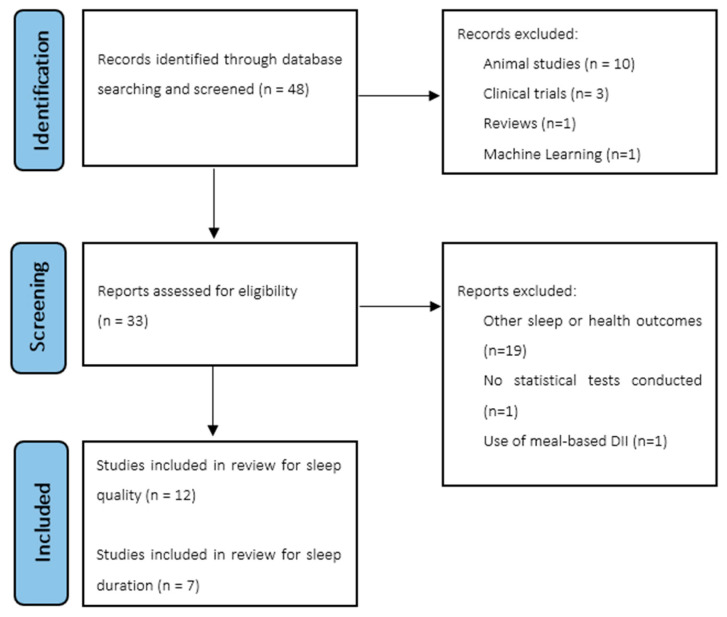
PRISMA flowchart.

We identified 45 publications and screened them for eligibility based on the inclusion and exclusion criteria. Thirty-two studies were excluded. Eleven and seven studies that met all the inclusion criteria were included in the current review for sleep quality and sleep duration, respectively (Figure 1).

### 3.2. DII and Sleep Duration

Seven studies assessed the association between sleep duration and dietary inflammation using the DII. Three studies assessed sleep duration by using objective methods (i.e., using actigraphy) [24,26,29]. Four studies assessed sleep duration subjectively using either the Pittsburgh Sleep Quality Index (PSQI) [19,20,33] or self-reported hours of sleep per night [21]. Summaries of all the studies are presented in Table 1.

Six studies were conducted in healthy adults across the lifespan [19,20,21,24,26,29,33]. Of these, two reported outcomes in younger participants [26,33], while four studies focused on middle-aged participants [19,20,21,24]. Across the six studies, only two studies reported an association between sleep duration and dietary inflammation, such that more inflammatory diets were associated with a shorter (less than 6 h) [19,21] and longer (more than 9 h) [21] sleep duration. 

One study assessed the correlation between sleep duration and dietary inflammation in pregnant participants with overweight or obesity and showed no association between sleep duration and dietary inflammatory scores [29].

### 3.3. DII and Sleep Quality

Twelve studies assessed the link between sleep quality (as measured by the Pittsburgh Sleep Quality Index, where higher scores indicate poor sleep quality [34]) and the DII. Summaries of all the studies are presented in Table 1.

Seven of these studies were conducted in healthy adults across the lifespan [19,20,23,24,26,30,33]. Studies that have been conducted mainly in young adults found null associations between sleep quality and the DII [19,26,30,33]. However, in middle-aged adults, Wirth, Fekedulegn, et al. (2022) [24] showed that more pro-inflammatory diets were correlated with higher improved subjective sleep quality. By using representative samples across age groups (but by mainly recruiting young-to-middle-aged adults), Godos, Ferri, Caraci, Cosentino, Castellano, Shivappa, et al. (2019) [20] showed that individuals in the highest quartile of the DII (i.e., individuals who follow more pro-inflammatory dietary patterns) were less likely to have adequate sleep quality and, similarly, Wang et al. (2022) [23] showed that more pro-inflammatory diets were linked with poor sleep quality (in individuals with poor sleep quality). 

The remainder of the studies were conducted in young-to-middle-aged unhealthy adults [18,22,25,27]. While studies conducted in overweight and/or obese individuals found significant associations between higher scores on the DII and poor sleep quality [18,22] (apart from Tabrizi and Farhangi, 2021 [28]), other studies showed no significant association between the DII and sleep quality in individuals with sleep apnea [27] and fibromyalgia [25].

### 3.4. Risk of Bias 

The quality of the studies included in this review was evaluated by one reviewer (PH) using the Agency for Healthcare Research and Quality (AHRQ) checklist [35]; Table 2. The 11-item AHRQ checklist has “yes”, “no”, or “unclear” classifications. The studies are classified as “high quality” (8–11 items with a “yes” response); moderate quality (4–7 items with a “yes” response); and “low quality” (0–3 items with a “yes” response). The applied quality appraisal revealed eleven studies to be of high quality, one study to be of moderate quality, and two studies to be of low quality. 

## 4. Discussion

In terms of sleep duration, small cross-sectional studies in healthy adults (*n* < 500), reported no significant correlation between DII scores and sleep duration [20,24,26,33]. Similar findings were reported in pregnant participants with overweight or obesity [29]. One study reported an association between higher DII scores and shorter sleep duration amongst university employees in Iran [19]; however, it is important to highlight that the majority of participants (i.e., 85% and above) reported a short sleep duration of less than 6 h, which may not represent the broader population [36]. Hence, this limitation raises concern for the generalizability of their findings. A large-scale survey (*n* > 30,000) conducted by Kase et al. (2021) [21], on the other hand, revealed that higher dietary inflammation scores, indicative of a pro-inflammatory diet, were associated with both shorter (less than 6 h) and longer (more than 9 h) sleep durations. Notably, this study utilized data from the National Health and Nutrition Examination Survey, where sleep duration was assessed through self-reported hours of sleep at night on weekdays or workdays. In contrast, the study by Behbahani et al. (2022) [19] utilized the Pittsburgh Sleep Quality Index (PSQI) to evaluate self-reported sleep duration, although specific details about variable information are not clarified in the study. Given that different sleep assessments (e.g., actigraphy, diary, and retrospective questionnaires) are known to yield different estimates of sleep duration [37], findings should be interpreted with caution. 

Taken together, the observed discrepancies across studies may be attributed to variations in study size, sample representation, and different methods used to assess sleep duration, with some studies relying on subjective self-report measures. Notably, studies involving both healthy and unhealthy participants that measured sleep objectively using actigraphy reported no significant link between sleep duration and dietary inflammatory scores [24,26,29]. This highlights the importance of standardized methodologies in future research.

In terms of sleep quality, small-scale (*n* < 430) cross-sectional studies in healthy adults did not show an association between sleep quality and DII [19,30,33] (apart from Wirth, Fekedulegn, et al., 2022 [24]). However, it is important to note that, in the Wirth, Fekedulegn, et al., 2022 study [24], participants (i.e., police officers) were exposed to several stressors that can affect sleep, including working night and evening shifts, shift changes, and higher depression and anxiety levels (which is in line with previous work showing health disparities in this population [38]). Hence, their inconsistent results could be attributable to these confounding factors. On the other hand, in large-cohort studies (*n* > 2000) with healthy adults, an association between reduced sleep quality and adherence to pro-inflammatory dietary patterns was found [20,23]. Therefore, null results reported above could be explained by the lack of power and sample representatives. Nevertheless, a correlation between reduced sleep quality and following pro-inflammatory dietary patterns may reflect the mediatory role of inflammation in the associations between diet quality and sleep quality. In fact, a previous study shows the mediating role of various inflammatory markers (such as platelet and neutrophil counts, CRP levels, and NLR) on the diet and sleep quality relationship in generally healthy older adults [8]. Hence, reducing dietary and/or circulating pro-inflammatory biomarkers via dietary interventions may offer a promising primary and/or alternative approach to improving sleep quality. 

Additionally, studies conducted in obese and/or overweight individuals showed an association between reduced sleep quality and adherence to pro-inflammatory dietary patterns [18,22] (apart from Tabrizi and Farhangi, 2021 [28]; in which the null finding could be driven by the different statistical analysis technique, namely, the structural equational modeling, used). However, a similar pattern of results was not observed in studies conducted in populations with pain-related disorders or sleep-related breathing disorders [25,27]. Given that (i) obesity and/or being overweight are states of low-grade systemic chronic inflammation [39] and (ii) systemic chronic inflammation (i.e., elevated pro-inflammatory biomarkers such as CRP) is associated with poor sleep quality [8], the results could reflect that the DII could be linked to sleep quality only in individuals with compromised inflammatory status. However, more research that elucidates the relationship between the DII and sleep quality in other metabolic conditions (such as non-communicable diseases) that are characterized by persistently high concentrations of circulating pro-inflammatory biomarkers is warranted. 

The biological mechanisms regarding the association between the DII score and sleep duration and sleep quality could involve (i) cytokine responses, (ii) the neuroendocrine and autonomic pathways that link sleep with the immune system, and (iii) the role of inflammatory peptides in the homeostatic regulation of sleep (for a review, please see [40]). However, it is also important to highlight that various sociodemographic factors (such as low socioeconomic status and reduced access to healthy food and healthcare) could also contribute to the DII and sleep association, as they have all been shown to be associated with poor dietary choices (hence leading to anti-inflammatory dietary patterns) and poor sleep outcomes [41,42].

There are various limitations of the current review. Firstly, the studies involved in this review had no standard way of utilizing the DII and/or PSQI, such that these measures were used in a categorical and/or continuous manner. Therefore, due to the heterogeneity of the extracted data, no quantitative meta-analysis could be performed. Secondly, FFQ, dietary recall, and PSQI measures are subject to response/recall bias, as both sleep outcomes and dietary intakes are known to be under-reported [43,44]. Thirdly, there was heterogeneity in terms of DII score calculations, such that the number of food parameters included for estimating the DII differed substantially across studies. Fourthly, seven studies were conducted in female-only samples, limiting the generalizability of the findings. Fifthly, it is important to note that the studies included in this review did not systematically account for factors that may contribute to poor sleep (such as caffeine intake, lack of exercise, poor diet, stress, etc.); hence, it is highly possible that the results could be driven by the impact of these factors on sleep duration and quality. Finally, due to the cross-sectional nature of the studies, causality cannot be assumed. 

## 5. Conclusions

The current review offers a thorough assessment of the literature on the association between sleep duration and quality and the DII and shows that pro-inflammatory diets may be associated with poor sleep outcomes (duration and quality). However, as the current literature is heterogenous, future studies are required to (i) replicate previous findings in large cohorts across age groups, utilizing all 45 food parameters that are required to estimate the DII, preferably by using objective sleep outcomes, and (ii) examine the potential benefits of adhering to anti-inflammatory diets on sleep outcomes and beyond. 

## Figures and Tables

**Table 1:** Summary of the studies involved in the review.

Authors	Participants	Design	Dietary Measures	Dependent Measures	Results: Sleep Duration	Results: Sleep Quality	Control Variables
[26]	427 (210 males) M_age_ = 27.6 SD_age_ = 3.8 Range_age_ = 21–35	Cross-sectional	24 h dietary recall	Objective sleep duration (actigraphy), PSQI sleep quality	NS	NS	BMI, waist-to-hip ratio, blood pressure, blood composition, gender, age, education, income, employment status, marital status, children, race, physical and sedentary activity (monitor), and scores on Eating Attitudes Questionnaire, Perceived Stress Scale, Social Approval, Social Desirability
[30]	293 females exclusively breastfeeding for ≤6 months Range_age_ = 25–45	Cross-sectional	Food frequency questionnaire (FFQ)	PSQI sleep quality		NS	Age, educational and occupational levels, number of babies and caregivers, level of social support
[29]	207 females with pre-pregnancy overweight or obesity M_age_ = 29.8 SD_age_ = 5.0 Range_age_ = 18–44	Cross-sectional (longitudinal)	24 h dietary recall	Objective sleep duration (actigraphy)	NS		Age, race, education, marital status, income, employment, insurance type, statis for Special Supplemental Nutrition Program for Women, Infants, and Children, number of children in the household, parity, vitamin intake, fast food intake, smoking status prior to pregnancy, scores on Medical Outcomes Study Social Support Survey, Perceived Stress Scale, Social Support for Diet, Social Support for Physical Activity, physical activity (monitor)
[24]	401 police officers (295 males) M_age_ = 41.5 SD_age_ = 6.7	Cross-sectional (longitudinal)	Food frequency questionnaire (FFQ)	Objective sleep duration and sleep quality (actigraphy), PSQI sleep quality	NS	More pro-inflammatory diets are associated with better sleep quality	Age, race, education, sex, sleep medication, tobacco and alcohol consumption, work history, years employment, BMI, waist circumference, systolic blood pressure, scores on Depression scale, Anxiety inventory, and Impact of events
[23]	5594 adults Age ≥ 30	Cross-sectional	Dietary survey NHANES 2005–2008	PSQI sleep quality		More pro-inflammatory diets are associated with poor sleep quality	
[19]	211 adults (103 males) Range_age_ = 18–50	Cross-sectional	Food frequency questionnaire (FFQ)	PSQI sleep duration, PSQI sleep quality	More pro-inflammatory diet associated with shorter sleep duration	NS	Energy intake
[22]	219 females with overweight or obesity Range_age_ = 17–58	Cross-sectional	Food frequency questionnaire (FFQ)	PSQI sleep quality		More pro-inflammatory diets are associated with poor sleep quality	Age, physical activity, energy, BMI
[18]	249 females with obesity M_age_ = 23.88 SD_age_ = 3.81 Range_age_ = 18–35	Cross-sectional	Food frequency questionnaire (FFQ)	PSQI sleep quality		More pro-inflammatory diets are associated with poor sleep quality	Energy intake (Model 1), age, education, physical activity, energy intake (Model 2)
[21]	30,121 (14,488 males) M_age_ = 47.19 SE_age_ = 0.26	Cross-sectional	Dietary survey NHANNES 2005–2016	Self-reported sleep duration: categories short <6 h), recommended (6–9 h), long (>9 h)	E-DII scores significantly higher in short and long sleep duration participants		Age, sex, BMI, race/ethnicity, education, marital status, chronic medical condition.
[33]	379 students (136 males) Range_age_ = 18–21	Cross-sectional	Food frequency questionnaire (FFQ)	PSQI sleep duration, PSQI sleep quality	NS	NS	Age, sex, nationality, marital status, living situation, income, smoking status, education, college type, BMI, and physical activity
[28]	278 females with obesity or overweight M_age_ = 31.40 SD_age_ = 10.89	Cross-sectional	Food frequency questionnaire (FFQ)	PSQI sleep quality		NS	
[25]	95 females with FMS diagnosis; 98 women (menopause status matched) controls	Cross-sectional	24 h dietary recall	PSQI sleep quality		NS	Age, menopausal status, and overall energy levels
[20]	2044 (804 males) Range_age_ = <30-≥70	Cross-sectional	Food frequency questionnaire (FFQ)	PSQI sleep duration, PSQI sleep quality	NS	Individuals who follow more pro-inflammatory dietary patterns are less likely to have adequate sleep quality	Age, sex, marital, educational, and occupational status, smoking and alcohol drinking habits, and physical activity level
[27]	296 Obstructive Sleep Apnea patients Range_age_ = 18–60	Cross-sectional	Food frequency questionnaire (FFQ)	PSQI sleep quality		NS	Sex, BMI, circumferences of waist and neck, poor sleep quality, physical activity, daytime sleepiness, diastolic blood pressure, alcohol consumption, energy intake, household income, fatigue, shiftwork

**Table 2:** Risk of bias assessment by using the Agency for Healthcare Research and Quality checklist [35].

	Q1	Q2	Q3	Q4	Q5	Q6	Q7	Q8	Q9	Q10	Q11	Total
[26]	+	+	-	-	?	+	+	+	+	+	+	8
[30]	+	+	-	+	+	+	+	-	+	+	+	9
[29]	+	+	-	+	+	+	+	+	NA	+	+	9
[24]	+	+	-	+	+	+	+	+	NA	+	+	9
[23]	+	+	+	+	+	+	+	+	+	+	?	10
[19]	+	+	-	-	+	+	+	+	+	+	?	8
[22]	+	+	-	-	?	+	+	+	NA	+	-	6
[18]	+	+	-	-	+	+	+	+	-	+	+	8
[21]	+	+	+	+	+	+	+	+	NA	+	+	10
[21]	+	?	-	+	?	+	+	+	+	-	-	6
[28]	+	?	+	+	?	+	+	+	NA	-	+	7
[25]	+	+	-	+	+	+	+	+	+	+	-	9
[20]	+	+	+	+	?	+	+	+	+	+	-	9
[27]	+	+	-	+	+	+	+	+	+	+	-	9

Please see the Supplementary Material for the questions included in the checklist. Green/+: Yes; Red/-: No; Amber/?: Unclear; NA: Blue/Not applicable. Total column denotes the number of Green/+ responses (≤3: Low quality; 4–7: Moderate quality; ≥8: High quality).

## Data Availability

Data is contained within the article or Supplementary Material.

## Supplementary Materials

The following supporting information can be downloaded at: https://www.mdpi.com/article/10.3390/nu16060890/s1.

## References

[1] Doherty R., Madigan S., Warrington G., Ellis J. (2019). Sleep and Nutrition Interactions: Implications for Athletes. Nutrients.

[2] Peuhkuri K., Sihvola N., Korpela R. (2012). Diet promotes sleep duration and quality. Nutr. Res..

[3] Sanlier N., Sabuncular G. (2020). Relationship between nutrition and sleep quality, focusing on the melatonin biosynthesis. Sleep Biol. Rhythm..

[4] St-Onge M.-P., Mikic A., Pietrolungo C.E. (2016). Effects of Diet on Sleep Quality. Adv. Nutr. Int. Rev. J..

[5] Godos J., Ferri R., Caraci F., Cosentino F.I.I., Castellano S., Galvano F., Grosso G. (2019). Adherence to the Mediterranean Diet is Associated with Better Sleep Quality in Italian Adults. Nutrients.

[6] Hepsomali P., Groeger J.A. (2021). Diet, sleep, and mental health: Insights from the UK Biobank study. Nutrients.

[7] Bonaccio M., Di Castelnuovo A., De Curtis A., Costanzo S., Persichillo M., Donati M.B., Cerletti C., Iacoviello L., de Gaetano G. (2014). Adherence to the Mediterranean diet is associated with lower platelet and leukocyte counts: Results from the Moli-sani study. Blood.

[8] Hepsomali P., Groeger J.A. (2022). Examining the role of systemic chronic inflammation in diet and sleep relationship. J. Psychopharmacol..

[9] Whalen K.A., McCullough M.L., Flanders W.D., Hartman T.J., Judd S., Bostick R.M. (2016). Paleolithic and Mediterranean Diet Pattern Scores Are Inversely Associated with Biomarkers of Inflammation and Oxidative Balance in Adults. J. Nutr..

[10] Wu P.-Y., Chen K.-M., Tsai W.-C. (2021). The Mediterranean Dietary Pattern and Inflammation in Older Adults: A Systematic Review and Meta-analysis. Adv. Nutr. Int. Rev. J..

[11] Koelman L., Rodrigues C.E., Aleksandrova K. (2022). Effects of Dietary Patterns on Biomarkers of Inflammation and Immune Responses: A Systematic Review and Meta-Analysis of Randomized Controlled Trials. Adv. Nutr. Int. Rev. J..

[12] Schwingshackl L., Hoffmann G. (2014). Mediterranean dietary pattern, inflammation and endothelial function: A systematic review and meta-analysis of intervention trials. Nutr. Metab. Cardiovasc. Dis..

[13] De Heredia F.P., Garaulet M., Gómez-Martínez S., Díaz L.E., Wärnberg J., Androutsos O., Michels N., Breidenassel C., Cuenca-García M., Huybrechts I. (2014). Self-reported sleep duration, white blood cell counts and cytokine profiles in European adolescents: The HELENA study. Sleep Med..

[14] Ferrie J.E., Kivimäki M., Akbaraly T.N., Singh-Manoux A., Miller M.A., Gimeno D., Kumari M., Smith G.D., Shipley M.J. (2013). Associations between change in sleep duration and inflammation: Findings on C-reactive protein and interleu-kin 6 in the Whitehall II Study. Am. J. Epidemiol..

[15] Lee H.-W., Yoon H.-S., Yang J.J., Song M., Lee J.-K., Lee S.-A., Choi J.-Y., Kang D. (2020). Association of sleep duration and quality with elevated hs-CRP among healthy Korean adults. PLoS ONE.

[16] Liu R., Liu X., Zee P.C., Hou L., Zheng Z., Wei Y., Du J. (2014). Association between sleep quality and C-reactive protein: Results from national health and nutrition examina-tion survey, 2005–2008. PLoS ONE.

[17] Shivappa N., Steck S.E., Hurley T.G., Hussey J.R., Hébert J.R. (2014). Designing and developing a literature-derived, population-based dietary inflammatory index. Public Health Nutr..

[18] Bazyar H., Javid A.Z., Behbahani H.B., Shivappa N., Hebert J.R., Khodaramhpour S., Zadeh S.K., Aghamohammadi V. (2021). The association between dietary inflammatory index with sleep quality and obesity amongst iranian female students: A cross-sectional study. Int. J. Clin. Pract..

[19] Behbahani H.B., Borazjani F., Sheikhi L., Amiri R., Angali K.A., Nejad S.B., Samadani M. (2022). The As-sociation between Diet Quality Scores with Sleep Quality among Employees: A Cross-Sectional Study. Ethiop. J. Health Sci..

[20] Godos J., Ferri R., Caraci F., Cosentino F.I.I., Castellano S., Shivappa N., Hebert J.R., Galvano F., Grosso G. (2019). Dietary Inflammatory Index and Sleep Quality in Southern Italian Adults. Nutrients.

[21] Kase B.E., Liu J., Wirth M.D., Shivappa N., Hebert J.R. (2021). Associations between dietary inflammatory index and sleep problems among adults in the United States, NHANES 2005–2016. Sleep Health.

[22] Setayesh L., Yarizadeh H., Majidi N., Mehranfar S., Amini A., Himmerich H., Casazza K., Mirzaei K. (2023). The negative relationship of dietary inflammatory index and sleeping quality in obese and overweight women. Int. J. Vitam. Nutr. Res..

[23] Wang L., Sun M., Guo Y., Yan S., Li X., Wang X., Hu W., Yang Y., Li J., Li B. (2022). The Role of Dietary Inflammatory Index on the Association Between Sleep Quality and Long-Term Cardiovascular Risk: A Mediation Analysis Based on NHANES (2005–2008). Nat. Sci. Sleep.

[24] Wirth M.D., Fekedulegn D., Andrew M.E., McLain A.C., Burch J.B., Davis J.E., Hébert J.R., Violanti J.M. (2022). Longitudinal and cross-sectional associations between the dietary inflammatory index and objectively and subjectively measured sleep among police officers. J. Sleep Res..

[25] Correa-Rodríguez M., Casas-Barragán A., González-Jiménez E., Schmidt-RioValle J., Molina F., Aguilar-Ferrándiz M.E. (2019). Dietary Inflammatory Index Scores Are Associated with Pressure Pain Hypersensitivity in Women with Fibromyalgia. Pain Med..

[26] Farrell E.T., Wirth M.D., McLain A.C., Hurley T.G., Shook R.P., Hand G.A., Hébert J.R., Blair S.N. (2023). Associations between the Dietary Inflammatory Index and Sleep Metrics in the Energy Balance Study (EBS). Nutrients.

[27] Lopes T.V., Borba M.E., Lopes R.V., Fisberg R.M., Paim S.L., Teodoro V.V., Zimberg I.Z., Araújo L.B., Shivappa N., Hébert J.R. (2019). Association between inflammatory potential of the diet and sleep parameters in sleep apnea patients. Nutrition.

[28] Tabrizi F.P.F., Farhangi M.A. (2021). Is there any mediatory association between health-related quality of life and eating behaviors to affect dietary inflammatory index (DII) among reproductive-aged women? A structural equation modeling approach. Nutr. Clin. Métabolisme.

[29] Wirth M.D., Liu J., Wallace M.K., McLain A.C., Turner-McGrievy G.M., Davis J.E., Ryan N., Hébert J.R. (2022). Dietary Inflammatory Index and sleep quality and duration among pregnant women with overweight or obesity. Sleep.

[30] Zou H., Sun M., Liu Y., Xi Y., Xiang C., Yong C., Liang J., Huo J., Lin Q., Deng J. (2022). Relationship between Dietary Inflammatory Index and Postpartum Depression in Exclusively Breastfeeding Women. Nutrients.

[31] Moher D., Shamseer L., Clarke M., Ghersi D., Liberati A., Petticrew M., Shekelle P., Stewart L.A., PRISMA-P Group (2015). Preferred reporting items for systematic review and meta-analysis protocols (prisma-p) 2015 statement. Syst. Rev..

[32] Shamseer L., Moher D., Clarke M., Ghersi D., Liberati A., Petticrew M., Shekelle P., Stewart L.A., PRISMA-P Group (2015). Preferred reporting items for systematic review and meta-analysis protocols (PRISMA-P) 2015: Elaboration and explanation. BMJ.

[33] Masaad A.A., Yusuf A.M., Shakir A.Z., Khan M.S., Khaleel S., Ismail L.C., Faris M.A.-I.E., Jahrami H.A., Shivappa N., Hebert J.R. (2021). Sleep quality and Dietary Inflammatory Index among university students: A cross-sectional study. Sleep Breath..

[34] Buysse D.J., Reynolds C.F., Monk T.H., Berman S.R., Kupfer D.J. (1989). The Pittsburgh Sleep Quality Index: A new instrument for psychiatric practice and research. Psychiatry Res..

[35] Rostom A., Dubé C., Cranney A., Saloojee N., Sy R., Garritty C., Sampson M., Zhang L., Yazdi F., Mama-ladze V. (2014). Celiac Disease: Summary. AHRQ Evidence Report Summaries.

[36] Yazdanpanah M.H., Farjam M., Naghizadeh M.M., Jedi F., Mohebi K., Homayounfar R. (2021). Sleep duration and anthropometric indices in an Iranian population: The Fasa PERSIAN cohort study. Sci. Rep..

[37] Matthews K.A., Patel S.R., Pantesco E.J., Buysse D.J., Kamarck T.W., Lee L., Hall M.H. (2018). Similarities and differences in estimates of sleep duration by polysomnography, actigraphy, diary, and self-reported habitual sleep in a community sample. Sleep Health.

[38] Hartley T.A., Burchfiel C.M., Fekedulegn D., Andrew M.E., Violanti J.M. (2011). Health disparities in po-lice officers: Comparisons to the U.S. general population. Int. J. Emerg. Ment. Health.

[39] Ellulu M.S., Patimah I., KhazáAi H., Rahmat A., Abed Y. (2017). Obesity and inflammation: The linking mechanism and the complications. Arch. Med. Sci..

[40] Irwin M.R. (2019). Sleep and inflammation: Partners in sickness and in health. Nat. Rev. Immunol..

[41] Alkerwi A., Vernier C., Sauvageot N., Crichton G.E., Elias M.F. (2015). Demographic and socioeconomic disparity in nutrition: Application of a novel Correlated Component Regression approach. BMJ Open.

[42] Groeger J.A., Hepsomali P. (2023). Social Deprivation and Ethnicity Are Associated with More Problematic Sleep in Middle-Aged and Older Adults. Clocks Sleep.

[43] Lauderdale D.S., Knutson K.L., Yan L.L., Liu K., Rathouz P.J. (2008). Self-reported and measured sleep duration: How similar are they. Epidemiology.

[44] Shim J.-S., Oh K., Kim H.C. (2014). Dietary assessment methods in epidemiologic studies. Epidemiol. Health.

