# Supplementary material for: Dietary Inflammatory Index, Sleep Duration, and Sleep Quality: A Systematic Review
Source: Nutrients. 2024 Mar 19;16(6):890. doi: 10.3390/nu16060890 (PMC10974932; doi:10.3390/nu16060890)
Supplement: Supplementary file 1 [file nutrients-16-00890-s001.zip › nutrients-2883733-SI.pdf]

Supplementary Material - Dietary Inflammatory Index, Sleep Duration and Quality: A Systematic Review

Christle Coxon [1], Jun Nishihira [2] and & Piril Hepsomali [3]

<u>The Agency for Healthcare Research and Quality (AHRQ) checklist questions (Rostom et al., 2014)</u>

Q1: Define source of information

Q2: List inclusion and exclusion criteria for subjects or refer to previous publications

Q3: Indicate whether subjects were consecutive if not population based. Whether subjects are representative of the average in the community?

Q4: Indicate time period used for identifying subjects

Q5: Indicate if evaluators of subjective components of study were masked to other aspects of the status of the participants

Q6: Is the examination method standard?

Q7: Describe any assessments undertaken for quality assurance purposes

Q8: Are the assessments and classification of caries index and BMI clearly stated and standard?

Q9: If any, explain any subject exclusions from analysis

Q10: Describe how confounding was assessed and/or controlled

Q11: Summarize patient response rates and completeness of data collection.

References

1. Rostom, A., Dubé, C., Cranney, A., Saloojee, N., Sy, R., Garritty, C., Sampson, M., Zhang, L., Yazdi, F., & Mamaladze, V. (2014). Celiac Disease: Summary. In: AHRQ Evidence Report Summaries. Rockville (MD): Agency for Healthcare Research and Quality (US); 1998-2005. 104. Available from: https://www.ncbi.nlm.nih.gov/books/NBK11885/
